# Muffin Technique Micrographic Surgery for Non-melanoma Skin Cancer

**DOI:** 10.3389/fmed.2020.637223

**Published:** 2021-01-21

**Authors:** Philip Surmanowicz, Arunima Sivanand, Amy X. Du, Muhammad N. Mahmood, Robert Gniadecki

**Affiliations:** ^1^Division of Dermatology, Faculty of Medicine and Dentistry, University of Alberta, Edmonton, AB, Canada; ^2^Department of Pathology, Faculty of Medicine and Dentistry, University of Alberta, Edmonton, AB, Canada

**Keywords:** surgical techniques, micrographic surgery, non-melanoma skin cancer, basal cell carcinoma, squamous cell carcinoma, excision

## Abstract

**Background:** Mohs micrographic surgery (MMS) is the gold standard treatment for high-risk facial non-melanoma skin cancer. However, patients' access to MMS is limited by cost. The muffin technique micrographic surgery (MTMS) is an alternative micrographic technique wherein the entire excised margin is evaluated post-operatively by a pathologist using paraffin-embedded material. Herein, we describe the implementation and the preliminary results of MTMS in an academic dermatology center.

**Objective:** To describe the MTMS and outline its efficacy and safety in a real-world clinical academic setting.

**Methods:** A retrospective chart review was conducted of all patients with basal cell carcinoma (BCC) and squamous cell carcinoma (SCC) who underwent MTMS at the University of Alberta Dermatology Center from June 2016 until July 2019.

**Results:** A total of 69 patients were included (64 BCCs and 5 SCCs). 68.1% of surgeries had clear margins following the first incision, 100% after second round re-excisions. There were no observed cases of tumor recurrence after a median 40 months of follow-up. There were no major adverse events or complications.

**Conclusions:** MTMS is a superior alternative to simple excision of skin cancer by providing full margin control and residual tumor mapping.

## Introduction

Surgical treatment of non-melanoma skin cancer (NMSC) comprises two major approaches: simple excision and micrographic surgery. The key difference is that simple excision is done with predetermined margins based on the perceived risk of relapse, whereas micrographic surgery prospectively examines the entire excised margins and re-excises any residual tumor tissue. However, the favorably lower risk of recurrence achieved with micrographic surgery comes with the disadvantage of a higher technical complexity of residual tumor mapping resulting in higher cost of treatment, which presents a limitation for its use as a first-choice therapeutic option in all instances.

Mohs micrographic surgery (MMS) is the most widespread micrographic technique for NMSC (1). The tumor is excised at a 45° angle, after which the specimen is flattened on slides, frozen, and then cryosectioned horizontally to visualize the lateral and deep borders in a single section. MMS is superior to simple excision, boasting exceptionally low rates of recurrence, especially for high-risk tumors (2, 3). However, MMS is costly and resource-intensive. A Mohs surgeon requires specialized training in the interpretation of histopathology slides, and a large surgical space is needed to accommodate several surgeries in parallel along with the bedside histology lab. Thus, the use of MMS is restricted to aggressive NMSC of the face, where a balance between radical excisions and cosmetic considerations are of the utmost importance. There have been attempts to replace the traditional MMS frozen tissue technique with dermatological ultrasound, optical coherence tomography, and confocal microscopy, but each of these approaches have failed at reliably detecting small areas of residual tumor (4–6).

**Figure 1:**
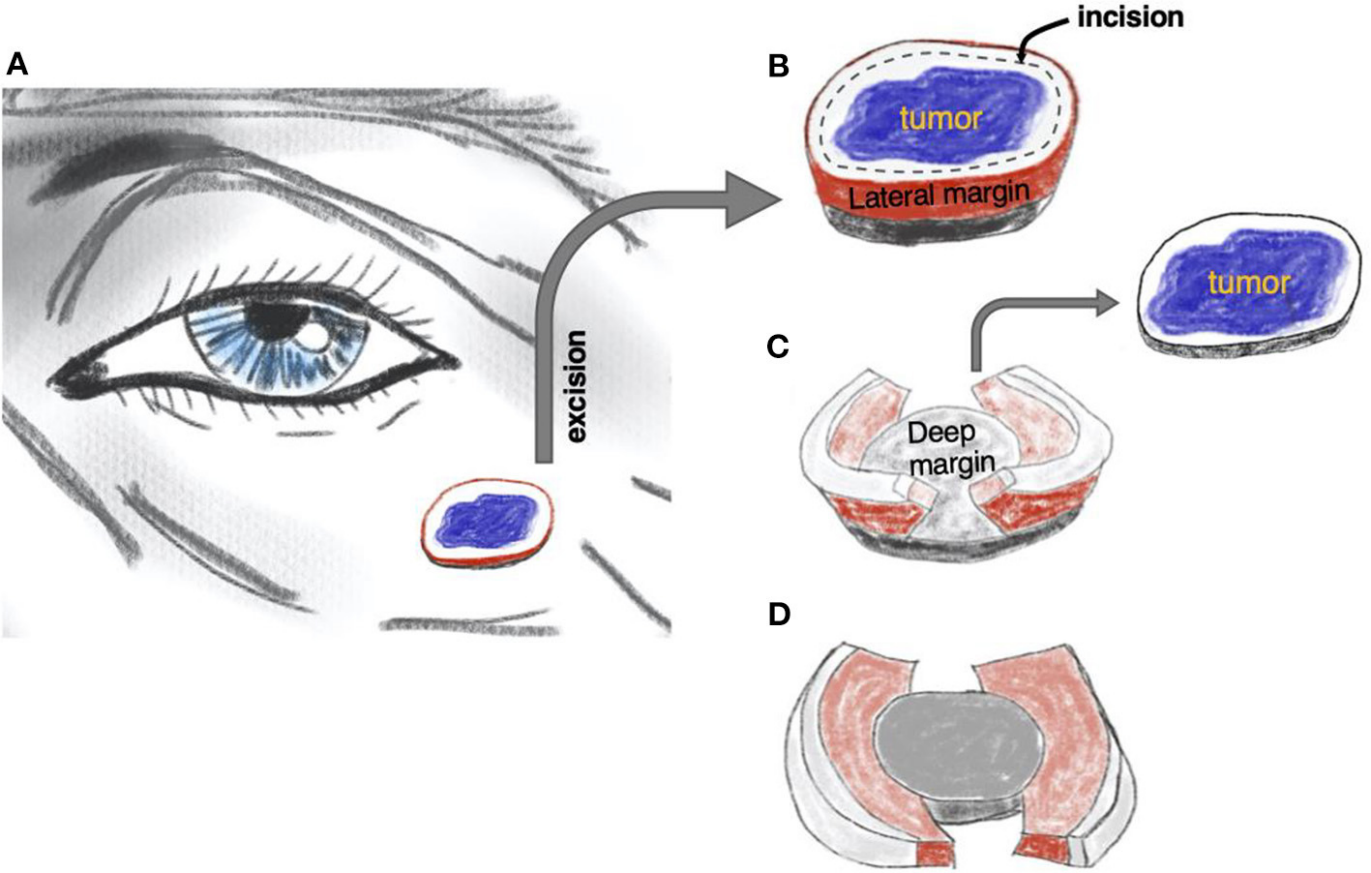
The principle of muffin technique micrographic surgery. The tumor is resected with a margin of healthy skin **(A)**. The excised specimen is then incised 1–2 mm from the edge of the tissue block to form the lateral margins and ≅1 mm over the floor to form the deep margins **(B)**. The central portion containing the tumor, the “muffin” itself, is removed **(C)** and the specimen is flattened to bring the lateral and deep margins, the “muffin paper,” into the same plane and color-coded for orientation **(D)**. The histological sections are cut after being formaldehyde fixed and paraffin embedded in a horizontal direction starting from the bottom of the specimen. Of note, larger soft tumors can be curetted before excision, as is often practiced in mohs micrographic surgery. See also Supplementary Video 1.

In 1984, Breuninger published a seminal description of the whole margin, micrographic “Tübingen torte” technique using paraffin-embedded specimens (7). This approach combined the efficiency of simple surgical excision with the advantages of micrographic surgery. Since then, the technique has evolved with several published permutations, culminating in 2006 with the so-called “muffin technique” (MTMS, muffin technique micrographic surgery) (8). In contrast to traditional MMS that involves incising at 45° angles to encircle the lesion and create a bowl-shaped specimen, the MTMS uses standard 90° angles to remove the tumor. Subsequently, the lateral and deep margins, the “muffin paper,” are prepared, color-coded, paraffin-embedded, and sent for horizontal sectioning similar to the approach in traditional MMS. The remaining central bulk tumor (the “muffin” itself) is sent for pathology in a separate container (see Figure 1 and Supplementary Video 1 for technical details). The following sectioning and histopathological examination is performed in a regular histopathology lab by a pathologist, which frees up the surgical team's time and space.

Compared to MMS, the drawback of MTMS is that the histological examination cannot be completed promptly during the surgery and that the surgeon must decide whether the defect should be left for later reconstruction or closed with the risk of additional surgery later in the case of residual tumor.

The MTMS has been successfully employed in Germany but remains relatively unknown in North America. Herein, we describe the implementation of MTMS in an academic dermatology center and report on resulting outcomes, efficacy, and safety.

## Materials and Methods

We conducted a retrospective cohort review of cases of basal cell carcinomas (BCC) and squamous cell carcinomas (SCC) excised by MTMS at the University of Alberta Dermatology Center between June 2016 and July 2019.Each patient underwent micrographic tumor excision by the MTMS as described in Figure 1 and Supplementary Video 1. All procedures were conducted or supervised by the same single practitioner who also has expertise with classic frozen tissue MMS technique (RG). All MTMShistopathology evaluations were performed by the same dermatopathologist (NM).

The diameters of the tumors and their subsequent excisional defects were measured using a sterile ruler along the greatest diameter (D). The areas (S) of both lesions were calculated by approximation to a circular shape:

$$\begin{array}{l} \text{S }=\text{ π }\times \;\left(1/2\text{D}\right) \end{array}$$

Statistical analyses were conducted using R software (Table 1). The chi-squared test was employed to generate *p*-values for observations of anatomical lesion location and tumor pathology data. Fisher's exact test was done in place of a chi-squared test for any comparison containing a group with <5 individuals, specifically when evaluating the diagnosis. The Kruskal-Wallis test was used to generate *p*-values for comparisons of tumor size and surgical defect diameter. A *p* < 0.05 was considered statistically significant.

**Table 1:** Demographics, disease characteristics, surgical details, and outcomes for the sample cohort after primary surgical excision (*n* = 69).

**Characteristics**	**Complete**	**Incomplete**
	**resection**	**resection**
Total patients, *n*	47	22
Mean age in years [Range]	69.0 [40–93]	66.5 [39–88]
Sex, *n* (%)		
Male	25 (53.2)	14 (63.6)
Female	22 (46.8)	8 (36.4)
Diagnosis, *n* (%)		
Basal cell carcinoma	42 (89.4)	22 (100)
Squamous cell carcinoma	5 (10.6)	0 (0)
Mean tumor size in mm (SD) [Range]	11.8 (6.4) [2–30]	12.4 (5.6) [3–25]
Anatomical lesion locations, *n* (%)		
High risk areas (H-zone)	28 (59.6)	17 (77.3)
Low risk areas	19 (40.4)	5 (22.7)
Pathology, *n* (%)		
High risk	14 (29.8)	7 (31.8)
Low risk	33 (70.2)	15 (68.2)
Margins involved following a single procedure, *n* (%)		
Deep	0 (0)	9 (40.9)
Lateral	0 (0)	6 (27.3)
Both lateral and deep	0 (0)	7 (31.8)
Mean surgical defect diameter in mm (SD) [Range]	19.3 (7.0) [6–40]	18.7 (6.5) [9–31]
Surgical closure used, n (%)		
Side to side	27 (57.4)	9 (40.9)
Advancement flap	9 (19.1)	6 (27.3)
Transposition flap	3 (6.4)	4 (18.2)
Rotation flap	4 (8.5)	0 (0)
Bilobed flap	1 (2.1)	2 (9.1)
Island pedicle flap	2 (4.3)	1 (4.5)
Secondary intention	1 (2.1)	0 (0)
Mean duration follow-up in days [Range]	833 [222–1,391]	892 [222–1,391]

*The differences between the complete and incomplete resection groups were not statistically significant when evaluating diagnosis, tumor size, anatomic lesion location, pathology, or surgical defect diameter*.

## Results

### Patient Characteristics

A total of 69 patients were included, 39 men and 30 women, comprising 64 BCCs (92.8%) and 5 SCCs (7.2%). The mean age of participants was 68.2 years (range: 39–93 years).

### Disease Characteristics

**Figure 2:**
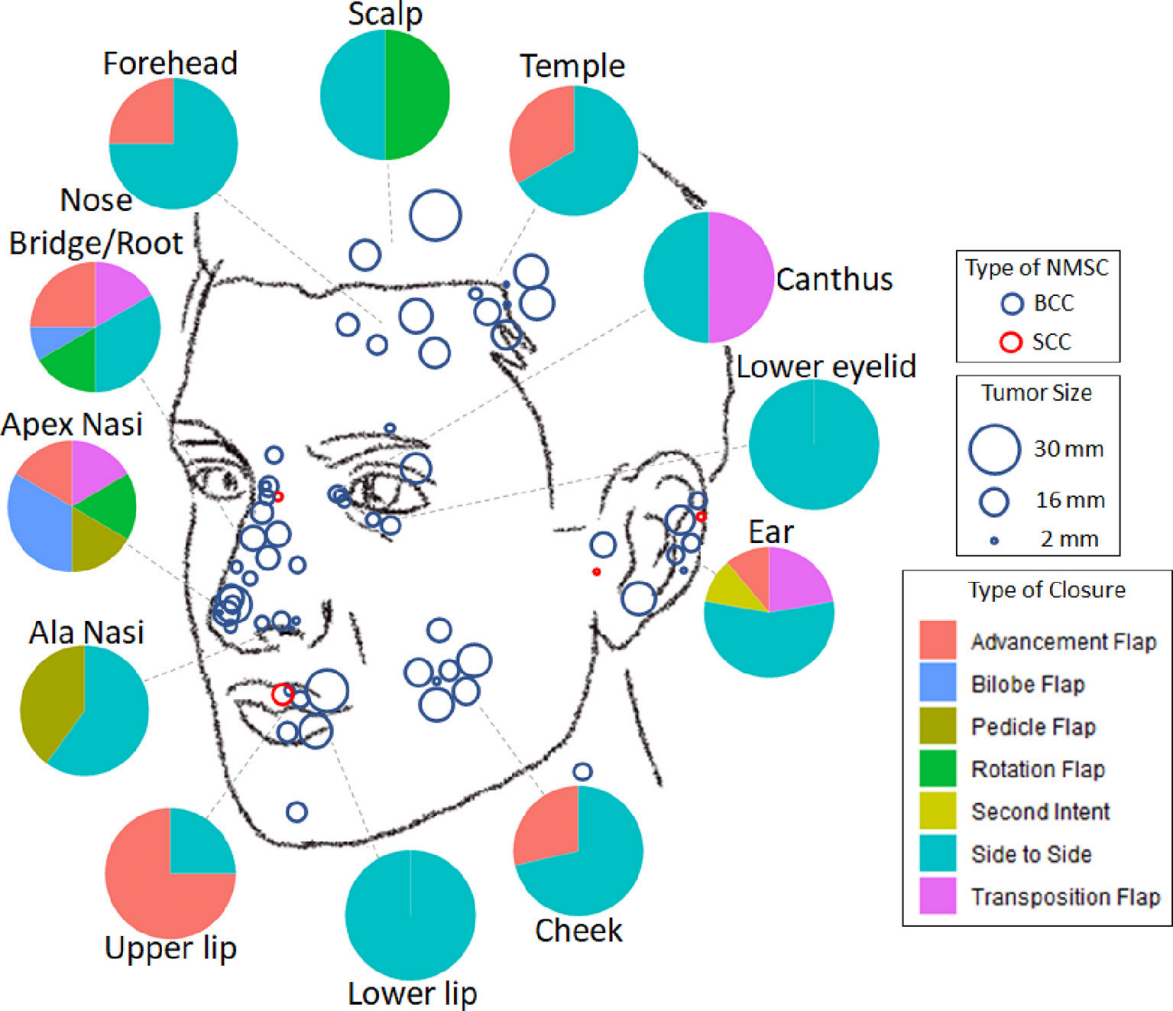
Distribution of facial non-melanoma skin cancer treated with muffin techniquemicrographic surgery. Blue—basal cell carcinoma, Red—squamous cell carcinoma; size of the circle is proportional to the size of the tumor. Pie diagrams illustrate frequencies of various types of closure at different areas of the face.

45 (65.2%) lesions were removed from “high-risk areas” of the nose (33.3%), the ear (13.0%), the periorbital region (10.1%), and the lips (8.7%) (Table 1, Figure 2). 21 (30.4%) lesions demonstrated “high-risk” pathology of infiltrative (20.3%), morphoeic (4.3%), micronodular (4.3%), and basosquamous (1.4%). The remaining 48 (69.6%) samples were noted as “low-risk” for pathology of nodular (62.3%), well-differentiated (4.3%), and superficial (2.9%). The majority, 61 (88.4%), of NMSCs cases were primary tumors with the remaining eight (11.59%) representing recurrences, of which three were due to prior incomplete excision. 11 (15.9%) tumors reached or exceeded a diameter of 20 mm.

### Micrographic Excision

The pathologist was able to evaluate the complete margin in all cases. Radical, margin-free tumor excision was accomplished with a single session in 47 (68.1%) cases. Deep margin involvement was observed in nine (13.0%) cases, lateral involvement in six (8.7%), and both deep and lateral margins in seven (10.1%). Diagnosis, tumor size, anatomical lesion location, pathology, and surgical defect diameter were not statistically significant when comparing the complete to the incomplete resection groups. Histologically clear margins were achieved in all cases of repeat excision.

MTMS proved modestly tissue-sparing compared to simple surgical excision with predetermined margins. Surgical defect sizes were 1.85-fold [95% confidence interval (CI): 1.66–2.04] the area of the tumors. By comparison, the predicted defect sizes using standard predetermined 4 mm margins for low-risk primary NMSC would be 1.96-fold the size of the tumors (95% CI: 1.79–2.14). Tissue sparing was more pronounced for high-risk tumors where the suggested 6 mm margins would yield defects 2.4-fold (95% CI: 1.35–3.54) larger than the tumor.

### Wound Closure

The choice of type of surgical defect closure depended primarily on the localization of the tumor (Figure 2). The majority (*n* = 36, 52.2%) of defects were closed side to side; 15 (21.7%) by advancement flap; seven (10.1%) by transposition flap; four (5.8%) by rotation flap; three (4.4%) by bilobed flap; three (4.4%) by island pedicle flap; and one lesion on the ear after excision of SCC was left to secondary intention healing.

### Outcomes and Safety

There were no cases of observed relapse following MTMS excision in our cohort. The mean duration of follow up was 815 days (range: 247–1,353), with 56 (81.2%) patients having exceeded 1 year, 38 (55.1%) 2 years, and 20 (29.0%) 3 years. Overall, there were no adverse events noted during or following surgery requiring unscheduled visits or rehospitalization. There were no cases of hypertrophic or keloid scarring, wound dehiscence, or infections.

## Discussion

Traditional MMS, with 1-year recurrence rates of 0.2% and favorable cosmetic results is considered a gold-standard efficacious and safe procedure for the treatment of BCCs and SCCs (2, 3, 9). However, MMS is costly, time-consuming, and in a not-for-profit health care delivery setting can only feasibly be employed for high-risk facial tumors (10, 11). Our experience with MTMS strongly suggests that this technique provides a robust alternative for the simple excision of tumors that do not fulfill the criteria for MMS or where there is lack of access to MMS.

The implementation of MTMS proved to be straightforward. The preparation of the “muffin” is simple and only takes 2–5 min of extra time. Apart from the tissue dyes, there is no additional procedural cost compared to simple excisions. Preparation of the pathology slides and their interpretation was uncomplicated and unambiguous. We believe that the directions provided in this paper alongside Supplementary Video 1 demonstrating tissue preparation will be sufficient for any dermatological surgeon to readily adopt this technique into everyday clinical practice. However, it should be noted that previous experience with MMS is advantageous and encouraged as it would aid the surgeon in determining the appropriate margins for primary tumor excision and increase confidence in tissue orientation and tumor mapping.

MTMS does not confer any disadvantages compared with simple excision of NMSC and, in our view, should replace the current breadloafing approach employed in the practice of dermatological surgery. MTMS was demonstrably tissue sparing (5.2% reduction in procedural defect size compared to excision with 4 mm margins and 22.0% reduction compared to excision with 6 mm margins). Similar to our experience with MMS, ≅ 70% of tumors were radically excised after a single procedure (12). MTMS offers micrographic mapping of residual tumor tissue which allows for a broader choice of closure techniques. With careful topographic tracing of the tumor, it is possible to map incompletely excised areas even after closures with asymmetric flaps such as bilobed or transposition flaps. Those residual areas can then be re-excised with minimal surgery once the pathology report is available, even weeks after the initial surgery.

We would also like to emphasize that the MTMS technique can be readily adapted for rapid margin assessment using cryosectioning. Rather than performing formalin fixation and paraffin embedment, the “muffin” can be flattened onto a standard microscope slide, frozen, and sectioned horizontally similarly to the traditional MMS technique. This approach would provide more reliable margin control than the typical intraoperative frozen section technique used by general and plastic surgeons which only examines <1% of the entire margin (vs. 100% in MMS and MTMS) (13–15).

**Figure 3:**
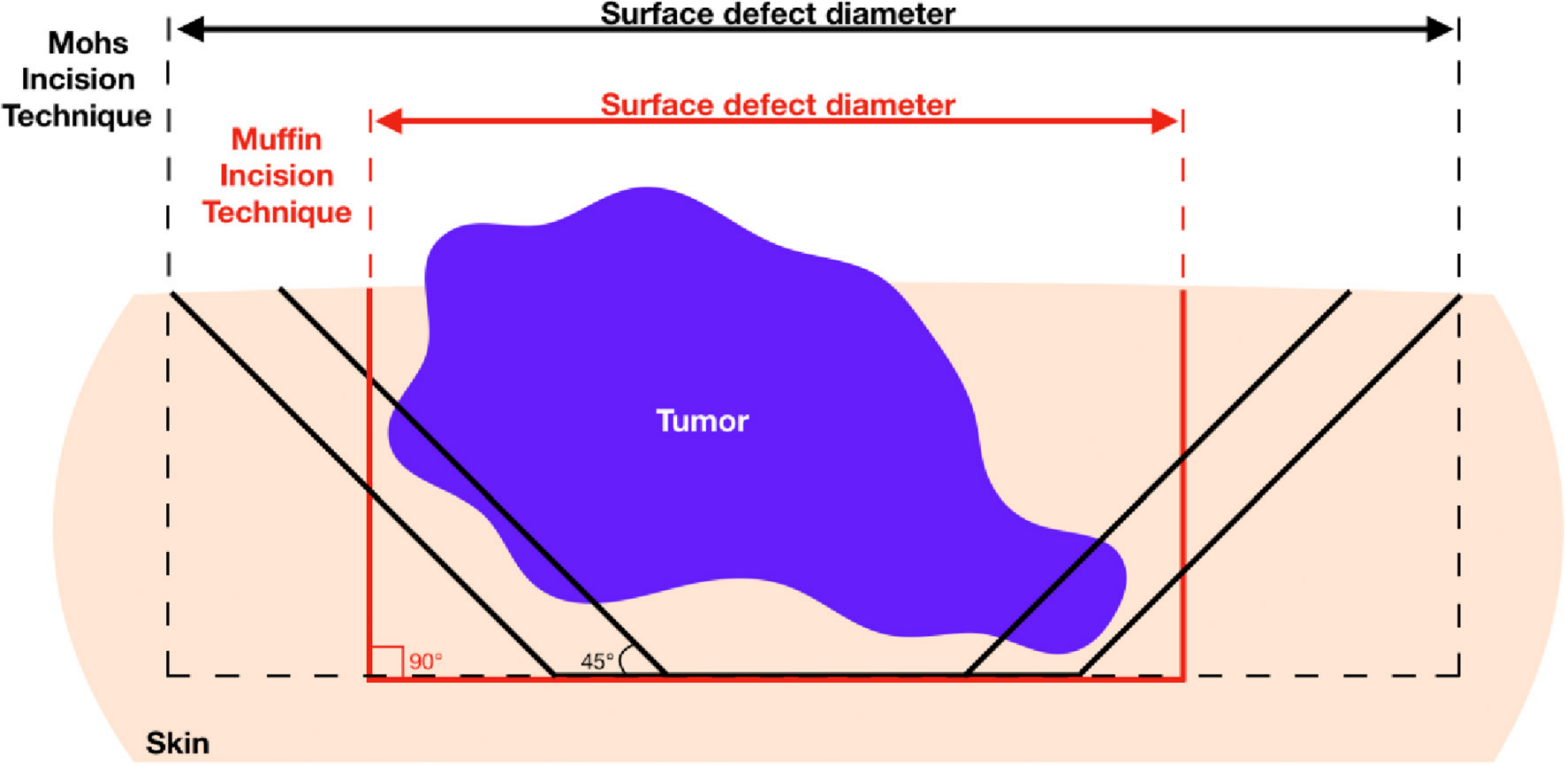
Advantage of 90° excision angle employed in muffin technique micrographicsurgery for obtaining tumor-free lateral margins and healthy skin conservation. Since most basal cell carcinomas grow asymmetrically producing deep finger-like projections, oblique incision may cut into the deeper lateral portions of the tumor yielding positive margins. In this case, repeat excision would be required ultimately increasing burden on patient and wound defect sizes.

Although the MTMS is not intended to replace frozen tissue MMS, the muffin approach offers several interesting advantages. Firstly, the quality of the prepared histology is superior to that which is achieved in MMS which may result in improved precision for the diagnosis of residual tumor. Artifacts due to tissue folding, imperfect flattening of the tissue, or presence of neoplastic “floaters” are common in MMS but are not encountered in paraffin-embedded MTMS (16–18). Secondly, the primary excision with a square 90° angle is favorable over the 45° angle traditionally employed for MMS because it diminishes the chance for false-positive lateral margins. As shown in Figure 3, oblique excisions may cut into the tumor in a situation where the square angle will yield a tumor-free margin. Moreover, square angle excisions allow for immediate closures without the need for edge de-beveling and may, therefore, be favorable for cosmetic results.

Three important limitations of our study are the limited number of patients evaluated with SCC, the short durations of follow-up, and the absence of direct comparison groups. Future studies should compare MTMS with patients treated with MMS and simple excision with predetermined borders. Taking into consideration that it required more than half a century to confirm Mohs initial data in a randomized clinical trial, stringent evidence-based data supporting the efficacy and safety of MTMS may unfortunately be difficult to obtain in a timely fashion (9, 19). In the meanwhile, cohort studies such as our own highlight the numerous advantages of MTMS as an alternative to simple excision of skin cancer.

## Data Availability Statement

The raw data supporting the conclusions of this article will be made available by the authors, without undue reservation.

## Ethics Statement

The studies involving human participants were reviewed and approved by Health Research Board of Alberta Cancer Committee under the ID HREBA.CC-19-0256. Written informed consent for participation was not required for this study in accordance with the national legislation and the institutional requirements.

## Author Contributions

PS and RG: study design. PS, AS, AD, MM, and RG: data collection. PS, AS, and RG: writing of the manuscript. All authors contributed to the article and approved the submitted version.

## Conflict of Interest

The authors declare that the research was conducted in the absence of any commercial or financial relationships that could be construed as a potential conflict of interest.
