# Supplementary material for: Muffin Technique Micrographic Surgery for Non-melanoma Skin Cancer
Source: Front Med (Lausanne). 2021 Jan 21;7:637223. doi: 10.3389/fmed.2020.637223 (PMC7859636; doi:10.3389/fmed.2020.637223)
Supplement: Supplementary file 1 [file Table_1.DOCX]

**Video S1.** Video demonstration of the muffin technique micrographic surgery sample preparation for histological evaluation.

https://drive.google.com/file/d/1RG7fO2yseDk5PYfJJMOkPRMn_zRy_1La/view?usp=sharing
